# Selected Uterine Immune Events Associated With the Establishment of Pregnancy in the Dog

**DOI:** 10.3389/fvets.2020.625921

**Published:** 2021-02-09

**Authors:** Miguel Tavares Pereira, Renata Nowaczyk, Rita Payan-Carreira, Sonia Miranda, Selim Aslan, Duygu Kaya, Mariusz P. Kowalewski

**Affiliations:** ^1^Institute of Veterinary Anatomy, Vetsuisse Faculty, University of Zurich (UZH), Zurich, Switzerland; ^2^Division of Histology and Embryology, Department of Biostructure and Animal Physiology, Faculty of Veterinary Medicine, Wroclaw University of Environmental and Life Sciences, Wroclaw, Poland; ^3^Mediterranean Institute for Agriculture, Environment (MED) and Department of Veterinary Medicine, University of Évora, Évora, Portugal; ^4^Animal and Veterinary Research Center (CECAV), University of Trás-os-Montes and Alto Douro, Vila Real, Portugal; ^5^Department of Obstetrics and Gynecology, Faculty of Veterinary Medicine, Near East University, Nicosia, Cyprus; ^6^Department of Obstetrics and Gynecology, Faculty of Veterinary Medicine, Kafkas University, Kars, Turkey

**Keywords:** dog (*Canis lupus familiaris*), immune system, uterus, early pregnancy, embryo-maternal communication

## Abstract

In the dog, implantation takes place at approximately 17 days of embryonal life and, while exposed to relatively high circulating progesterone concentrations, embryos presence is required for the formation of decidua. Furthermore, a balance between pro- and anti-inflammatory responses in conceptus-maternal communication is crucial for the onset of pregnancy. Strikingly, the understanding of such immune mechanisms in canine reproduction is still elusive. Here, canine uterine samples from pre-implantation (day 10–12, E+) and corresponding non-pregnant controls (E–), implantation (day 17, Imp) and post-implantation (day 18–25, Post-Imp) stages of pregnancy were used to investigate the expression and localization of several immune-related factors. The most important findings indicate increased availability of *CD4, MHCII, NCR1, IDO1, AIF1, CD25, CCR7*, and *IL6* in response to embryo presence (E+), while *FoxP3* and *CCL3* were more abundant in E– samples. Implantation was characterized by upregulated levels of *FoxP3, IL12a, ENG*, and *CDH1*, whereas *CD4, CCR7, IL8*, and *-10* were less represented. Following implantation, decreased transcript levels of *TNFR1, MHCII, NCR1, TLR4, CD206, FoxP3*, and *IL12a* were observed concomitantly with the highest expression of *IL6* and *IL1*β. MHCII, CD86, CD206, CD163, TNFα, IDO1, and AIF1 were immunolocalized in macrophages, CD4 and Nkp46 in lymphocytes, and some signals of IDO1, AIF1, and TNF-receptors could also be identified in endothelial cells and/or uterine glands. Cumulatively, new insights regarding uterine immunity in the peri-implantation period are provided, with apparent moderated pro-inflammatory signals prevailing during pre-implantation, while implantation and early trophoblast invasion appear to be associated with immunomodulatory and rather anti-inflammatory conditions.

## Introduction

The uterine mucosal immune system plays an important role during maternal recognition and establishment of pregnancy. This is accomplished by maintaining a balance between the defense against pathogens and the tolerance toward the allogeneic sperm and semiallogeneic embryo, adapting thereby to different pregnancy-associated events (e.g., implantation, placentation, parturition) and contributing to tissue remodeling (1–3). This balance relies on the complex population of resident immune cells, composed of, e.g., macrophages, natural killer (NK) cells, B and T lymphocytes, regulated by local and systemic signaling, including endocrine insults (1, 2, 4, 5), and changing during the progression of pregnancy. This, i.e., regards the polarization of uterine macrophages. Thus, as shown e.g., in humans, pre-implantation is marked by a predominant presence of M1 (pro-inflammatory) macrophages, transitioning to a mixed, M1/M2, population during trophoblast attachment and a predominant presence of M2 macrophages following placentation [reviewed in (6)]. While the role of all of the different immune cell populations in the uterus is not fully understood, some, like the NK are crucial in the onset of pregnancy. In the human and mouse uterus they are required for the formation of highly invasive (hemochorial) deciduate placenta [reviewed in (7, 8)]. NK cells are the most prevalent immune cell population in the uterus in these species and are involved in the regulation of trophoblast invasion, playing key roles in the remodeling of spiral arterioles during the formation of decidual tissue [reviewed in (8)]. However, despite presenting a deciduate endotheliochorial placenta, no such mechanisms are known for the dog, nor has the composition of the immune system been thoroughly studied in this species.

Furthermore, translational research from other species to the dog is limited by the canine species-specific decidualization mechanisms as a part of the peculiar reproductive physiology. Thus, lacking an active luteolytic event, non-pregnant bitches present a physiological pseudopregnancy that lasts frequently longer than pregnancy, with luteal P4 circulating levels similar to those observed in pregnant animals [reviewed in (9)]. Additionally, canine oocytes require oviductal maturation to reach fertilization competence, while implantation and development of a decidual endotheliochorial placenta start around days 17–18 after fertilization (10–12). Despite the high P4 levels observed during this period, no spontaneous decidualization can be observed in non-pregnant bitches, as the presence of the implanting blastocyst is needed to induce the differentiation of maternal stromal cells into decidual cells [reviewed in (13)]. Nevertheless, while the presence of an embryo-derived antiluteolytic signal is not needed in this species, cross-talk between the embryo and maternal tissue is still required for the establishment of pregnancy. Indeed, the presence of free-floating blastocysts has been associated with changes in the uterine transcriptome indicating modulation of, e.g., extracellular matrix components and immune system-related factors (14–16).

However, with regard to the involvement of the immune system in the establishment of pregnancy, available information for the dog pales in contrast to what is known for other species with a deciduate placenta. Canine early pregnancy is associated with increased circulating levels of acute phase and heat shock proteins that, due to their high sensitivity to the health status of the animal, have limited use as markers of pregnancy [reviewed in (13)]. As for embryo-induced changes, earlier qualitative analyses of the pre-implantation uterine immune milieu in pregnant and pseudopregnant bitches described differences in the presence of factors like CD4, CD8, INFγ, TNFα, and some interleukins (17, 18). In a later study from our group, the presence of free-floating embryos was associated with the predicted activation of signaling pathways involving IL1, IL10, toll-like receptor (TLR) and NFκB, and increased expression of chemokines and other immune regulators (14). As for the period of implantation and placentation, the available information is limited to the observed increased availability of leukemia inhibitory factor (LIF) and macrophage colony-stimulating (MCS) factor in placentation sites, in contrast with early pregnancy (17, 19). Furthermore, while immunohistochemical description of CD3^+^ T lymphocytes, macrophages and B lymphocytes is available for non-pregnant bitches (20), nothing is known regarding the uterine immune population of pregnant animals.

Consequently, while the immune system plays an important role in the onset of pregnancy, there is only scarce information available for the dog. To address this knowledge gap, the aim of this study was to characterize factors constituting the uterine immune milieu during the early stages of canine pregnancy, i.e., during the free-floating embryo stage (pre-implantation), at the time of implantation (day 17) and during placental formation (post-implantation). We investigated the expression and/or localization of twenty four different immune factors that serve as markers of different immune cells subsets and/or are involved in immune regulation. In addition, gene expression of six factors involved in tissue growth and remodeling was also evaluated.

## Materials and Methods

### Tissue Collection and Preservation

Uterine- and utero-placental samples from 27 (*n* = 27) healthy, mixed breed bitches aged 2–8 years old were allotted to the present work. All samples had been used before and details on animal manipulation and determination of pregnancy status were described in (14, 21–23). Briefly, animals were monitored for the onset of spontaneous ovulation by vaginal cytology and progesterone (P4) measurements. Health condition of the animals was evaluated by routine clinical examination. P4 levels were determined by radioimmunoassay, as previously described (24). Ovulation was considered to have occurred when circulating levels of P4 exceeded 5 ng/ml, and bitches were mated 2–3 days later (time needed for oocyte maturation and completion of the first meiotic division). Day of mating was considered day 0 of pregnancy. Samples were collected by ovariohysterectomy at different pregnancy stages: pre-implantation (days 8–12, before embryo apposition), at the day of implantation (Imp, day 17, *n* = 5) and during early placentation (post-implantation stage, Post-Imp, days 18–25, *n* = 5). During pre-implantation, early pregnancy was confirmed by uterine flushing and recovery of embryos (E+, *n* = 9). Animals in which no embryo was recovered between days 8–12 were considered not pregnant (E-, *n* = 8). Following surgery, retrieved uterine samples (including all histological layers) were immediately trimmed of connective tissues and washed in cold PBS. From Imp and Post-Imp groups, implantation/placentation sites were collected (the Post-Imp stage included the early utero-placental formation). For RNA analysis, samples were immersed in RNAlater (Ambion Biotechnology GmbH, Wiesbaden, Germany) for 24 h at 4°C and then stored at −80°C until used for RNA isolation. For histological analysis, samples were fixed for 24 h in 10% neutral phosphate-buffered formalin, washed with PBS, dehydrated in a graded ethanol series and embedded in the paraffin equivalent HistoComp (Vogel, Giessen, Germany).

Animal experiments were carried out in accordance with animal welfare legislation and were approved by the responsible ethics committee of the Justus-Liebig University Giessen, Germany (permits no. II 25.3-19c20-15c GI 18/14 and VIG3-19c-20/15 GI 18,14), and of the University of Ankara, Turkey (permits no. Ankara 2006/06 and 2008-25-124). Samples from day 17 of pregnancy were collected at the “Hospital Veterinário do Baixo Vouga”, Portugal, after informed consent of the owners (22).

### Total RNA Isolation, High Capacity Reverse Transcription, Pre-amplification of cDNA, and Semi-quantitative Real-Time TaqMan PCR (qPCR)

Total RNA was isolated using the TRIzol reagent (Invitrogen, Carlsbad, CA, USA) following the manufacturer's instructions and concentration and purity were assessed with a NanoDrop 2000C spectrophotometer (ThermoFisher scientific AG Reinach, Switzerland). The elimination of possible genomic DNA contamination was performed using the RQ1 RNA-free DNase Kit (Promega, Dübendorf, Switzerland) following the instructions provided by the manufacturer. Reverse transcription (RT) and pre-amplification of cDNA were performed by applying the High Capacity cDNA Reverse Transcription Kit (Applied Biosystems by ThermoFisher Scientific, Foster City, CA, USA), using 10 ng total RNA as starting material. Next, the TaqMan PreAmp Master Mix Kit (Applied Biosystems) was applied following the supplier's protocols and as previously described (25). Briefly, all used predesigned commercially available TaqMan systems (obtained from Applied Biosystems) and self-designed primers and 6-carboxyfluorescein (6-FAM) and 6-carboxytetramethylrhodamine (TAMRA) labeled probes (ordered from Microsynth AG, Balgach, Switzerland), were pooled. Afterwards, cDNA from each sample was mixed with PreAmp Master Mix and pooled TaqMan assays and samples were amplified in an Eppendorf Mastercycler (Vaudax-Eppendorf AG, Basel, Switzerland). A complete list of the predesigned TaqMan systems and self-designed primers and 6-FAM and TAMRA probes used is presented in Table 1.

**Table 1:** List of gene symbols, corresponding gene names, and TaqMan systems used for semi-quantitative real time qPCR.

**Gene**	**Name**	**Accession numbers**	**Primer sequence**		**Product length (bp)**
*MHCII*	Major histocompatibility complex II	NM_001011723.1	Forward	5′-GGA GAG CCC AAC ATC CTC ATC-3′	90
			Reverse	5′-GGT GAC AGG GTT TCC ATT TCG-3′	
			TaqMan probe	5′-TCG ACA AGT TCT CCC CAC C-3′	
*CD206/MCR1*	Cluster of differentiation 206/mannose receptor C-Type 1	XM_005617091.3	Forward	5′-GGC AGG AAG ATT GTG TCG TCA T-3′	108
			Reverse	5′-TGG GCT GGG TTT GAG ATT TC-3′	
			TaqMan probe	5′-TGG GCA GAT CGA GCC TGC GAG-3′	
*NCR1*	Natural cytotoxicity triggering receptor 1	NM_001284448.1	Forward	5′-CTG GGA TCA CAC TGC CCA TAA T-3′	103
			Reverse	5′-CCT CTT CCT GCA AAG CCA GTA-3′	
			TaqMan probe	5′-CTT TCC TGG TCC TGA TGG CCC TCA-3′	
*IL1β*	Interleukin 1 beta	NM_001037971.1	Forward	5′-TGC CAA GAC CTG AAC CAC AGT-3′	97
			Reverse	5′-CTG ACA CGA AAT GCC TCA GAC T-3′	
			TaqMan probe	5′-CAT CCA GTT GCA AGT CTC CCA CCA GC-3′	
*IL6*	Interleukin 6	AF275796.1	Forward	5′-AAA GAG CAA GGT AAA GAA TCA GGA TG-3′	124
			Reverse	5′-GCA GGA TGA GGT GAA TTG TG-3′	
			TaqMan probe	5′-ACT CCT GAC CCA ACC ACA GAC GCC A-3′	
*IL8/CXCL8*	Interleukin 8/ C-X-C motif chemokine ligand 8	NM_001003200.1	Forward	5′-CCA CAC CTT TCC ATC CCA AA-3′	114
			Reverse	5′-CCA GGC ACA CCT CAT TTC CA-3′	
			TaqMan probe	5′-CTG AGA GTG ATT GAC AGT GGC CCA CAT TGT-3′	
*TNFα*	Tumor necrosis factor alpha	NM_001003244	Forward	5′-TGC CCT TCC ACC CAT GTG-3′	96
			Reverse	5′-AGG GCT CTT GAT GGC AGA GA-3′	
			TaqMan probe	5′-CCC ACA CCA TCA GCC GCT TCG-3′	
*TNFR1*	Tumor necrosis factor receptor 1	XM_849381	Forward	5′-TGT GTG GCT GCA GGA AGA AC-3′	114
			Reverse	5′-GCT TCT CTT GGC AGG AGA TCT-3′	
			TaqMan probe	5′-ACT CCA CCC TCT GCC TCA ATG GCA-3′	
*TNFR2*	Tumor necrosis factor receptor 2	XM_005617982	Forward	5′-CCA GCA GAG CGA GTA CTT CGA-3′	95
			Reverse	5′-TCG AGG TCT TGG TGC AGA AGA-3′	
			TaqMan probe	5′-CAT GTG TCC CCC TGG CTC CCA C-3′	
*IDO1*	Indolamin 2,3-dioxygenase 1	XM_532793.5	Forward	5′-TGA TGG CCT TAG TGG ACA CAA G-3′	116
			Reverse	5′-TCT GTG GCA AGA CTT TTC GA-3′	
			TaqMan probe	5′-CAG CGC CTT GCA CGT CTG GC-3′	
*AIF1*	Allograft inflammatory factor 1	XM_532072.5	Forward	5′-CGA ATG CTG GAG AAA CTT GGT-3′	107
			Reverse	5′-TGA GAA AGT CAG AGT AGC TGAAGG T-3′	
			TaqMan probe	5′-TCC CCA AGA CCC ATC TGG AGC TCA A-3′	
*GAPDH*	Glyceraldehyde-3-phosphate dehydrogenase	AB028142.1	Forward	5′-GCT GCC AAA TAT GAC GAC ATC A-3′	75
			Reverse	5′-GTA GCC CAG GAT GCC TTT GAG-3′	
			TaqMan probe	5′-TCC CTC CGA TGC CTG CTT CAC TAC CTT-3′	
*CD163*	Cluster of differentiation 163		Pre-designed assay from Applied Biosystems, Prod.No. Cf02627321_m1		
*CD4*	Cluster of differentiation 4		Pre-designed assay from Applied Biosystems, Prod.No. Cf02627842_m1		
*CD8*	Cluster of differentiation 8		Pre-designed assay from Applied Biosystems, Prod.No. Cf02627888_m1		
*CD25/IL2Ra*	Cluster of differentiation 25/interleukin 2 receptor alpha		Pre-designed assay from Applied Biosystems, Prod.No. Cf02623133_m1		
*FoxP3*	Forkhead Box P3		Pre-designed assay from Applied Biosystems, Prod.No. Cf02741703_m1		
*IL10*	Interleukin 10		Pre-designed assay from Applied Biosystems, Prod.No. Cf02624264_m1		
*IL12a*	Interleukin 12		Pre-designed assay from Applied Biosystems, Prod.No. Cf02628398_m1		
*TGFβ*	Transforming growth factor 1 beta		Pre-designed assay from Applied Biosystems, Prod.No. Cf02623324_m1		
*CCL3*	C-C motif chemokine ligand 3		Pre-designed assay from Applied Biosystems, Prod.No. Cf02671956_m1		
*CCL13*	C-C motif chemokine ligand 13		Pre-designed assay from Applied Biosystems, Prod.No. Cf02622470_mH		
*CCR7*	C-C motif chemokine receptor 7		Pre-designed assay from Applied Biosystems, Prod.No. Cf02654980_m1		
*TLR4*	Toll-like receptor 4		Pre-designed assay from Applied Biosystems, Prod.No. Cf02622203_g1		
*IGF1*	Insulin-like growth factor 1		Pre-designed assay from Applied Biosystems, Prod.No. Cf02627846_m1		
*IGF2*	Insulin-like growth factor 2		Pre-designed assay from Applied Biosystems, Prod.No. Cf02647136_m1		
*ENG*	Endoglin		Pre-designed assay from Applied Biosystems, Prod.No. Cf02658400_m1		
*CDH1*	Cadherin-1/epithelial cadherin (E-cadherin)		Pre-designed assay from Applied Biosystems, Prod.No. Cf02624268_m1		
*ECM2*	Extracellular matrix protein 2		Pre-designed assay from Applied Biosystems, Prod.No. Cf02641132_m1		
*MMP2*	Matrix metalloperoteinase 2		Pre-designed assay from Applied Biosystems, Prod.No. Cf02741675_m1		
*β-ACTIN*	Beta-actin		Pre-designed assay from Applied Biosystems, Prod.No. Cf03023880_g1		
*PPIA/Cyclophilin*	Peptidylprolyl isomerase A		Pre-designed assay from Applied Biosystems, Prod.No. Cf03986523_gH		

The expression of the 29 selected target genes was investigated by real-time TaqMan PCR. The construction of self-designed primers and probes was based on published coding sequences (CDS). For genes where only predicted CDS were available (i.e., CD206 and NCR1), products were commercially sequenced (Microsynth) to confirm the specificity of amplicons. Efficiency values of PCR reactions were validated to ensure approximately 100% as previously described (25, 26). The protocols used for sample preparation and semi-quantitative real-time TaqMan PCR were published previously (25–27). TaqMan PCR was run with FastStart Universal Probe Master (ROX, Roche Diagnostics AG, Switzerland) and 5 μl pre-amplified cDNA. Reactions were run in duplicate in an automated ABI PRISM 7500 Sequence Detection System (Applied Biosystems). Autoclaved water and minus-RT controls were used instead of cDNA as negative controls, and relative quantification of gene expression was performed with the comparative Ct method (ΔΔCt), as previously described (26, 27). Values were calibrated to average expression in E– samples and normalized with the expression of reference genes. In preliminary experiments, the expression of three potential reference genes (*GAPDH*, β*-ACTIN* and *CYCLOPHILIN*) was evaluated in all used samples and their stability values were calculated using the online tool RefFinder (28). β*-ACTIN* and *GAPDH* were selected as more stable than *CYCLOPHILIN* and used as reference genes for ΔΔCt evaluation.

### Immunohistochemical Staining

Immunohistochemical (IHC) detection of 12 protein targets for which commercial canine cross-reacting antibodies were available (listed in Table 2) was performed using the standard indirect immunoperoxidase method, following our previously described protocol for canine tissues (27, 29). Briefly, formalin-fixed and paraffin-embedded tissue samples were cut in 2–3 μm thick sections, mounted on microscope slides (SuperFrost; Menzel-Glaeser, Braunschweig, Germany), deparaffinized and rehydrated. Slides were then heated in 10 mM citrate buffer (pH 6.0) in a microwave oven for antigen retrieval and endogenous peroxidase activity was quenched with 0.3% hydrogen peroxide diluted in methanol. Non-specific binding sites were blocked with 10% horse or goat serum (depending on the secondary antibody used), and samples were incubated overnight at +4°C. Dilutions of the selected primary antibodies are described in Table 2. Samples were then incubated with biotin-labeled secondary antibodies (horse anti-goat IgG BA-9500, goat anti-rabbit IgG BA1000 or horse anti-mouse IgG BA-2000, all purchased from Vector Laboratories Inc., Burlingame, CA, USA), diluted 1:100 in immunohistochemistry buffer (0.8 mM Na_2_HPO_4_, 1.47 mM KH_2_PO4, 2.68 mM KCl, and 137 mM NaCl, containing 0.3% Triton X; pH 7.2–7.4), followed by the avidin-peroxidase Vectastain ABC kit (Vector Laboratories Inc.). Peroxidase activity was detected using a liquid DAB + substrate kit (Dako Schweiz AG, Baar, Switzerland). Contrast staining was performed with hematoxylin and slides were mounted with Histokit (Assistant, Osterode, Germany). Evaluation of primary antibodies specificity was performed by replacing the primary antibody with a non-immune IgG from the same species and at the same concentration (isotype control, rabbit IgG I-1000, goat IgG I-5000, and mouse IgG I-2000, all from Vector Laboratories Inc.). Slides were evaluated qualitatively using a Leica DMRXE light microscope with a Leica DFC425 camera (Leica Microsystems, Wetzlar, Germany). Identification of macrophages and lymphocytes was performed by observing positive staining against specific factors, in some cases following staining of consecutive slides, and morphological characterization of cells following descriptions available in the literature (30, 31).

**Table 2:** List of antibodies and corresponding dilutions used in immunohistochemical staining.

**Target protein**	**Product reference and manufacturer**	**Dilution**
MHCII	ORB101661 (Biorbyt, Cambridge, UK)	1:200
CD86	ORB49101 (Biorbyt, Cambridge, UK)	1:400
Nkp46	ORB157934 (Biorbyt, Cambridge, UK)	1:400
CD4	AB125711 (Abcam, Cambridge, UK)	1:400
CD8	AB101500 (Abcam, Cambridge, UK)	1:100
TNFα	AB6671 (Abcam, Cambridge, UK)	1:200
TNFR1	AB19139 (Abcam, Cambridge, UK)	1:200
TNFR2	AB15563 (Abcam, Cambridge, UK)	1:200
IDO1	LS-C174759 (LSBio, Seattle, WA, USA)	1:50
AIF1	LS-B2403 (LSBio, Seattle, WA, USA)	1:400
CD206	SC-376108 (Santa Cruz Biotecnology Inc., Santa Cruz, CA, USA)	1:400
CD163	DB045 (DB Biotech, Kosice, SK)	1:400

### Statistical Analysis

Statistical evaluation of real-time TaqMan PCR was performed using the software GraphPad 2.06 (GraphPad Software Inc, San Diego, CA, USA). To evaluate the differences between the analyzed groups (i.e., in response to the presence or absence of embryos in the pre-implantation period, and between pre-implantation, implantation, and post-implantation stages) parametric one-way analysis of variance (ANOVA) followed by Tukey-Kramer multiple comparisons post-test were applied. Furthermore, the comparison between CD4 and CD8 expression in each experimental group was performed with an unpaired two-tailed Student's *t*-test. Numerical results for relative gene expression are presented as mean ± standard error of the mean (SEM). *P* < 0.05 was considered statistically significant.

## Results

**Figure 1:**
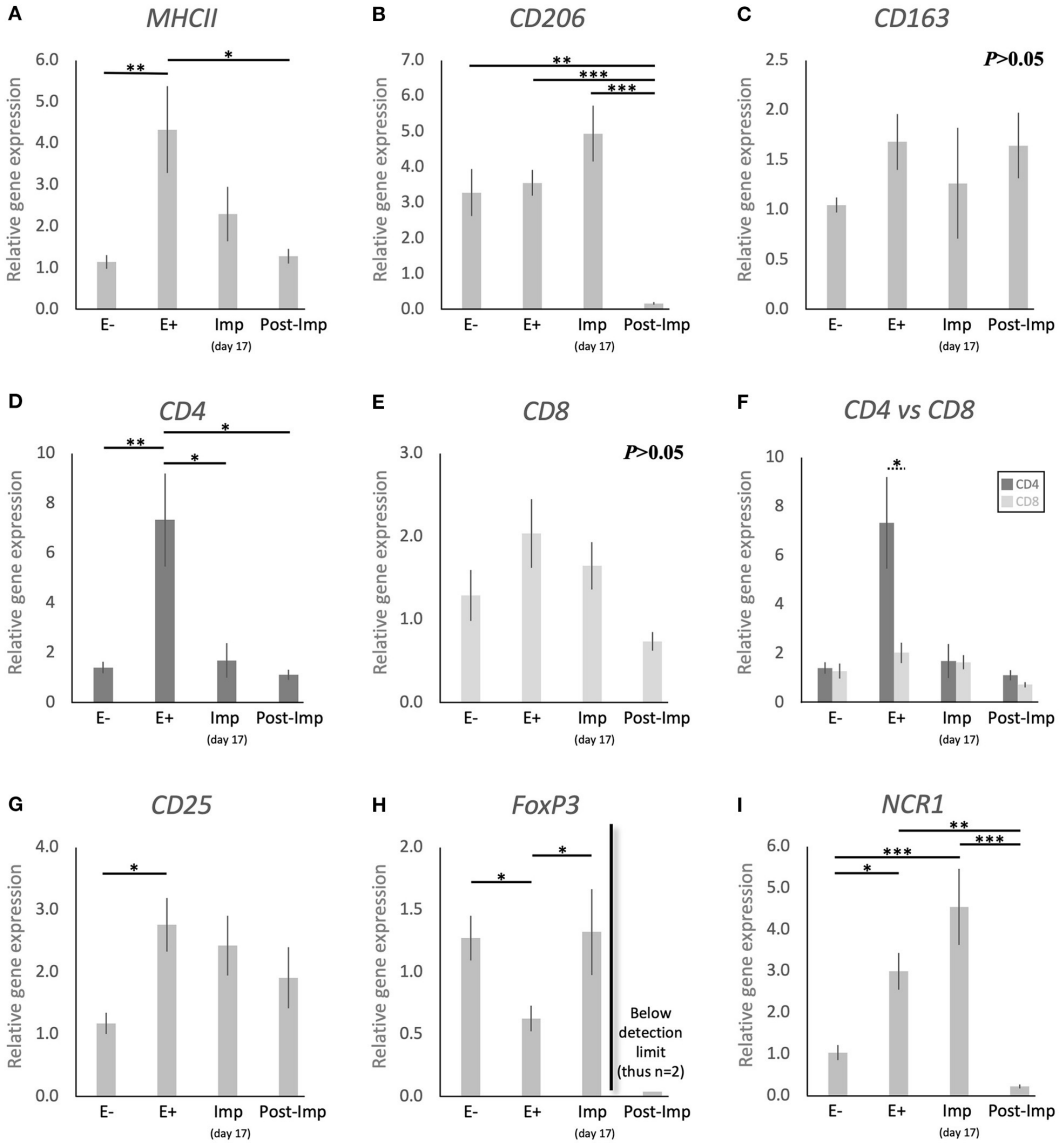
Relative gene expression of selected immune cell markers in the canine early pregnant uterus. Relative gene expression as determined by semi-quantitative real time (TaqMan) PCR (mean ± SEM). **(A–E,G–I)** One-way ANOVA was applied to test the effects of time (pregnancy stage) on gene expression revealing: *P* = 0.005 for *MHCII, P* < 0.0001 for CD206, *P* = 0.2 for CD163, *P* = 0.0032 for *CD4, P* = 0.06 for *CD8, P* = 0.02 for *CD25, P* = 0.01 for *FoxP3* and *P* < 0.0001 for *NCR1*. In the case of *P* < 0.05, the analysis was followed by a Tukey-Kramer multiple comparisons post-test. **(F)** Comparison of relative gene expression between CD4 and CD8 was evaluated by applying Student's unpaired two-tailed *t*-test at each investigated stage. Bars with asterisks differ at: *P < 0.05, **P < 0.01, ***P < 0.001.

With the aim of characterizing the presence and localization of several subsets of macrophages and lymphocytes in the early pregnant canine uterus, several relevant immune system markers, described in Table 3, were evaluated. Furthermore, the expression of several cytokines, immune regulators, and factors involved in tissue growth and remodeling was also assessed. The expression of the 29 target genes was detectable in samples from all experimental groups, although the transcript abundance of some of them at specific stages (indicated in Figures) was low and, sometimes, below detection limits. Among these were *FoxP3* and *IL12a* in samples from the Post-Imp stage (Figures 1H, **3E**, respectively) and *CCL13* on the day of implantation (day 17, **Figure 3K**). In addition, specific staining was obtained with all tested antibodies in all evaluated samples.

**Table 3:** Surface markers selected for identification of macrophage and lymphocyte subsets.

**Macrophage phenotype**	**Selected markers**
M1	↑MHCII, CD86, CD4
M2a	CD206
M2b	↓MHCII, CD86
M2c	CD163
**T lymphocyte subpopulation**	**Selected markers**
Tc (cytotoxic)	CD8
Th (helper)	CD4, CD25
Treg (regulatory)	CD4, CD25, FoxP3
NK (natural killer)	Nkp46 (NCR1)

*Selection of investigated markers was performed based on current literature (6, 32, 33, 36-40). (↑ = high expression, ↓ = low expression)*.

### Detection of Macrophages and Lymphocytes Markers

To investigate the presence of different macrophage phenotypes in the canine uterus during the establishment of pregnancy, the expression of *MHCII, CD206*, and *CD163* was evaluated. Pregnancy status (E+ vs. E–) and/or stage, significantly affected the expression of transcripts encoding for *MHCII* (*P* < 0.01) and *CD206* (*P* < 0.0001), but no significant changes were observed in the expression of *CD163* (*P* > 0.05, Figure 1C). The presence of embryos in the early pregnant uterus prior to implantation (E+) was associated with a higher expression of MHCII, in contrast to its non-pregnant counterparts (E–, *P* < 0.01, Figure 1A). This was not the case for *CD206*, for which the presence of embryos had no effect on the transcript levels (Figure 1B). However, considering the progression of pregnancy, the expression of *MHCII* and *CD206* was the lowest post-implantation (Post-Imp) when compared with E+ for *MHCII* (*P* < 0.05, Figure 1A) or all previous stages for *CD206* (*P* < 0.001, Figure 1B).

The different subsets of T lymphocytes were assessed by evaluating the uterine availability of transcripts encoding for *CD4, CD8, CD25, FoxP3*, and *NCR1* (that encodes for NKp46). Significant changes in the expression of *CD4* (*P* < 0.01), *CD25* (*P* < 0.05), *FoxP3* (*P* < 0.01), and *NCR1* (*P* < 0.0001) were observed in relation to the presence/absence of pregnancy and/or its stage, while *CD8* did not differ significantly (*P* > 0.05) across all analyzed groups (Figure 1). In the early pre-implantation period (days 8–12, E+), the presence of an embryo was associated with increased expression of *CD4* (*P* < 0.01, Figure 1D), *CD25* (*P* < 0.05, Figure 1G), and *NCR1* (*P* < 0.05, Figure 1I), compared with E- samples, while *FoxP3* was downregulated (*P* < 0.01, Figure 1H). Regarding pregnancy stage, *CD4* expression was also the highest during pre-implantation (E+), in contrast with the later stages, i.e., Imp and Post-Imp, when its uterine expression was significantly suppressed (*P* < 0.05, Figure 1D). In addition, *CD4* expression at the E+ stage was significantly higher than *CD8* (*P* < 0.05, Figure 1F). As for *FoxP3*, although the presence of embryos had a suppressive effect on its expression, its expression increased during Imp (*P* < 0.05, Figure 1H). Interestingly, following implantation the levels of *FoxP3* mRNA were significantly suppressed and fell below detection limits in most of the samples (Figure 1H). Similarly, the expression of *NCR1* decreased significantly after implantation (Post-Imp), compared with E+ and Imp (*P* < 0.01 and *P* < 0.001, respectively, Figure 1F).

**Figure 2:**
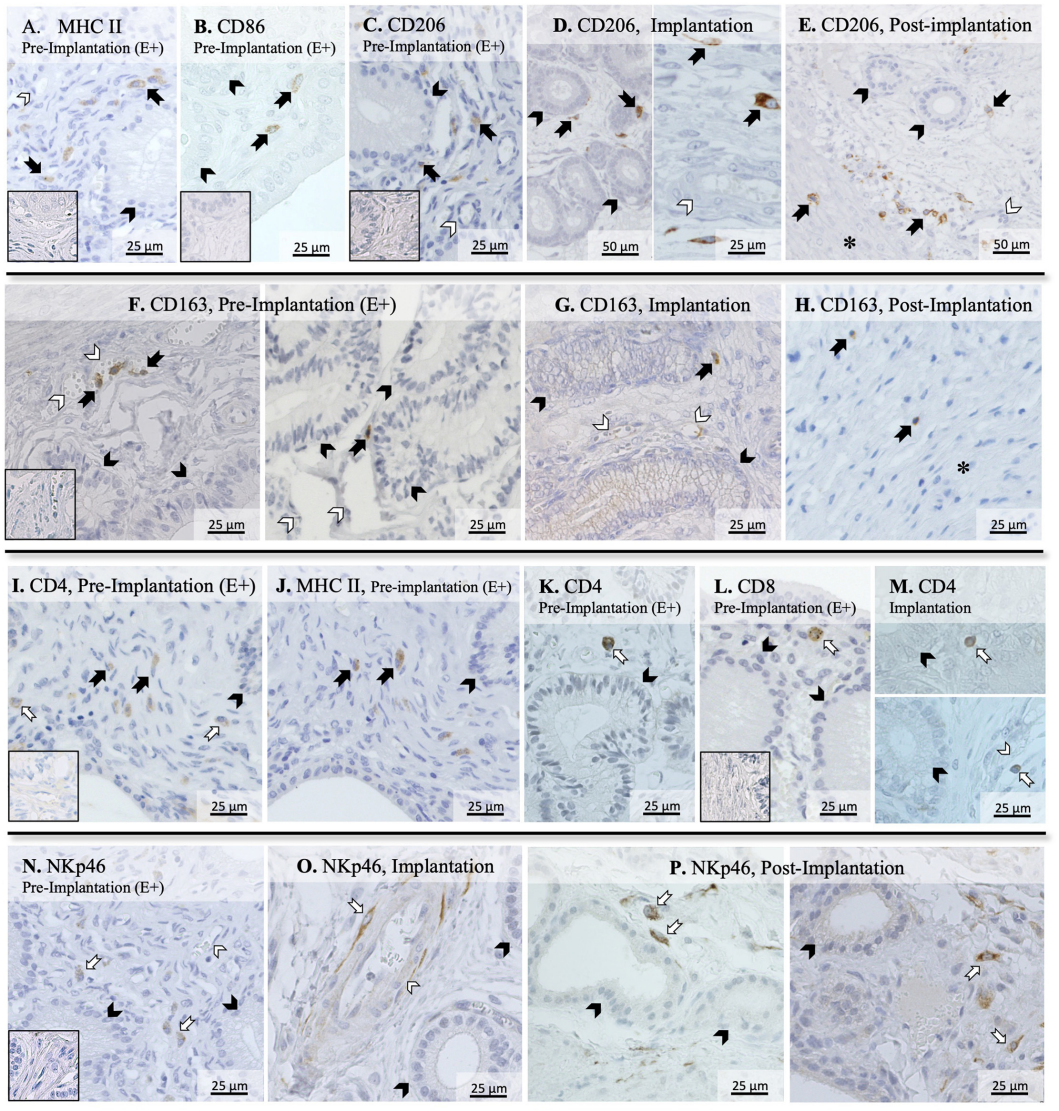
Immunolocalization of selected markers of macrophages and lymphocytes in the canine uterus. Immunohistochemical detection of MHCII, CD86, CD206, CD163, CD4, CD8, and NKp46 (encoded by *NCR1*) at selected stages during early pregnancy. MHCII (**A)** and CD86 **(B)** were localized in individual macrophages distributed in the superficial layer of the endometrium during pre-implantation. **(C–E)** CD206 signals were observed in macrophages localized in the superficial layer of the endometrium during pre-implantation, around uterine glands **(C)**. At the time of implantation, CD206-positive macrophages were present around uterine glands **(D**, left panel**)**, and scattered in the endometrium during implantation **(D**, right panel**)**, and post-implantation period **(E)**. Isolated cells could also be identified in the myometrium in Post-Imp samples **(E)**. **(F–H)** CD163 was expressed by macrophages detected in the superficial layer of the endometrium during pre-implantation **(F)** and implantation **(G)**. At post-implantation, single CD163-positive macrophages were localized in deep layers of the endometrium, close to the myometrium **(H)**. To differentiate CD4-expressing macrophages (MHCII^+^) and lymphocytes (MHCII^−^), consecutive slides were stained against CD4 **(I)** and MHCII **(J)**. Both CD4 **(K)** and CD8-positive lymphocytes **(L)** were localized in the superficial layer of the endometrium during pre-implantation. During implantation **(M)**, individual CD4^+^ lymphocytes were identified in endometrial superficial layer (top panel) and within blood vessels (bottom panel). Nkp46 (NK cells) were localized in the superficial layer of the endometrium **(N)** and around blood vessels **(O)** during pre-implantation and implantation stages. In samples from post-implantation stage **(P)**, NK cells were identified around superficial (left panel) and deep uterine glands (right panel). (solid arrow = macrophages; open arrow = lymphocytes; closed arrowhead = uterine gland; open arrowhead = blood vessel; asterisk = myometrium). No staining was observed in the isotype controls [inset in **(A–C,F,I,L,N)**].

To further evaluate the immune infiltrate in the canine uterus during the peri-implantation period, the immunolocalization of factors selected as markers of different immune cell subsets was also evaluated by IHC. Regarding factors expressed by macrophages, some MHCII positive cells were observed in the superficial layer of the endometrium of pregnant animals during the pre-implantation stage (Figure 2A), with a similar localization pattern observed for CD86 (Figure 2B). Likewise, CD206-positive signals were observed in macrophages mainly localized in superficial endometrial layers during pre-implantation (E+, Figure 2C). However, their localization appeared to change in subsequent stages of pregnancy, as cells expressing CD206 could be found scattered throughout the different layers of the endometrium and around deep uterine glands during implantation (Figure 2D) and in Post-Imp samples (Figure 2E). Isolated CD206-positive cells could also be identified in the myometrium in the different pregnancy stages (represented on Figure 2E). As for CD163, sporadic positive cells were observed in the superficial layer of the endometrium in pre-implantation (E+, Figure 2F) and Imp (Figure 2G) samples, while single cells identified as macrophages were present in the deep layers of the endometrium during post-implantation (Figure 2H). The differentiation between CD4-positive lymphocytes (MHCII-negative) and macrophages (MHCII-positive) was based on cell morphology and staining of consecutive slides against CD4 and MHCII (Figures 2I,J). CD4-positive lymphocytes were observed within the connective tissue in the superficial layer during the pre-implantation stage (Figures 2I,K). Additionally, scarce cells staining positive to CD8 could also be found in the same region of the endometrium (Figure 2L). At the time of implantation, lymphocytes expressing CD4 were mainly localized in the superficial layer of the endometrium (Figure 2M, top panel), with some being localized within blood vessels (Figure 2M, bottom panel). Moreover, staining against NKp46 was observed in numerous cells localized mainly at the surface layer of the endometrium and around blood vessels in pre-implantation (E+) and Imp samples (Figures 2N,O). However, during the post-implantation stage, NK cells were observed not only around superficial uterine glands (Figure 2P, left panel), but also around deep uterine glands (Figure 2P, right panel).

### Cytokines and Other Immune Regulators

**Figure 3:**
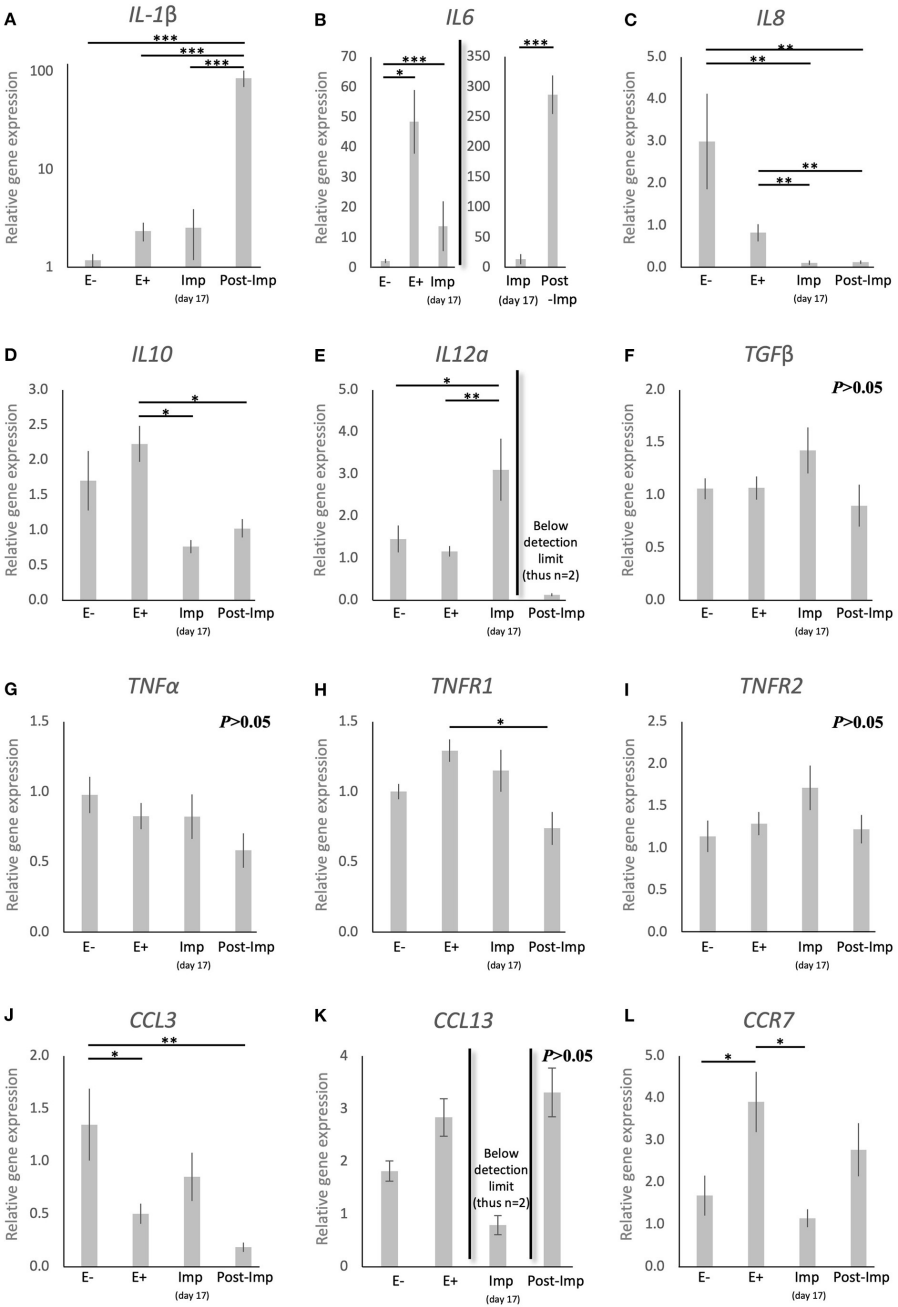
Relative gene expression of selected cytokines in the canine uterus. **(A–L)** Relative gene expression as determined by semi-quantitative real time (TaqMan) PCR (mean ± SEM). One-way ANOVA was applied, revealing: *P* < 0.0001 for *IL1*β, *P* < 0.0001 for *IL6, P* < 0.0001 for *IL8, P* = 0.01 for *IL10, P* = 0.02 for *IL12a, P* = 0.16 for *TGF*β, *P* = 0.19 for *TNF*α, *P* = 0.02 for *TNFR1, P* = 0.19 for *TNFR2, P* = 0.006 for *CCL3, P* = 0.083 for *CCL13* and *P* = 0.01 for *CCR7*. In the case of *P* < 0.05, this was followed by a Tukey-Kramer multiple comparisons post-test. Bars with asterisks differ at: *P < 0.05, **P < 0.01, ***P < 0.001.

To further characterize the immune milieu during the early stages of canine pregnancy, the expression of different cytokines was evaluated, including members of the TGF and TNF families, chemokines, and their receptors (Figure 3). The presence of the embryo and/or establishment of pregnancy were related to changes in the expression of *IL1*β (*P* < 0.0001), *IL6* (*P* < 0.0001), *IL8* (*P* < 0.0001), *IL10* (*P* < 0.01), *IL12a* (*P* < 0.05), *TNFR1* (*P* < 0.05), *CCL3* (*P* < 0.01), and *CCR7* (*P* < 0.01). In contrast, *TGF*β (Figure 3F), *TNF*α (Figure 3G), and *TNFR1* (Figure 3I) were not significantly affected (*P* > 0.05) by time in any of the analyzed groups. Furthermore, although no significant changes in the expression of *CCL13* were observed between the E–, E+, and Post-Imp groups (*P* > 0.05), its expression was apparently strongly suppressed at the time of implantation, being below detection limits in several samples (Figure 3K). The exposure of the uterus to embryos (E+) was associated with increased expression of *IL6* (*P* < 0.05, Figure 3B) and *CCR7* (*P* < 0.05, Figure 3L), when compared with E– samples, while the availability of *CCL3* transcripts was significantly lower in E+ (*P* < 0.05) and Post-Imp samples (*P* < 0.01, Figure 3J). Implantation was associated with the highest availability of *IL12a* (*P* < 0.01, Figure 3E), whereas its abundance was severely affected in the Post-Imp stage as it could not be detected in several samples (Figure 3E). This was different from what was observed for *IL1*β and *IL6* transcripts. Their strongly increased expression was observed during early placentation compared with the Imp stage (*P* < 0.001, Figures 3A,B, respectively). In contrast, early pregnancy was characterized by diminishing *IL8* levels, decreasing continuously following the onset of pregnancy, during implantation (Imp) and early placental development (Post-Imp) (*P* < 0.01, Figure 3C). Similar effects were observed for *IL10*, which was significantly reduced following the attachment of embryos and Post-Imp (*P* < 0.05, Figure 3D). Finally, *TNFR1* was downregulated during the progression of early pregnancy, being significantly lower in Post-Imp (*P* < 0.05, Figure 3H) compared with pre-implantation (E+) samples. As for *CCR7*, following its upregulated levels induced by embryo presence, its uterine levels were significantly suppressed during implantation (*P* < 0.05, Figure 3L).

**Figure 4:**
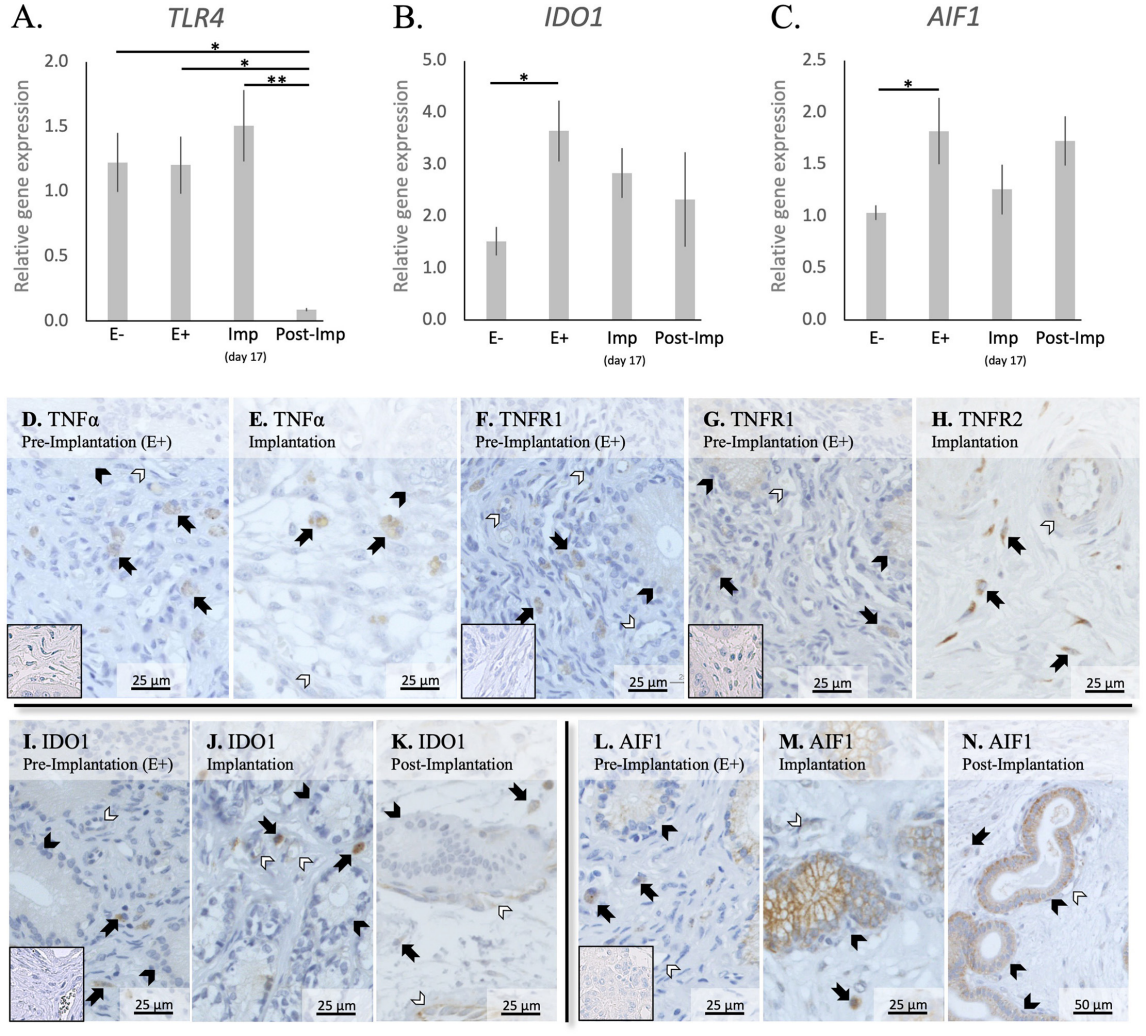
Relative gene expression and localization of TLR4, IDO1 and AIF1 in the canine uterus. **(A–C)** Relative gene expression as determined by semi-quantitative real time (TaqMan) PCR (mean ± SEM). One-way ANOVA was applied to test the variation among the investigated groups, revealing: *P* = 0.0033 for *TLR4, P* = 0.03 for *IDO1*, and *P* = 0.04 for *AIF1*. In the case of *P* < 0.05, this was followed by Tukey-Kramer multiple comparisons post-test. Bars with asterisks differ at: *P < 0.05, **P < 0.01. **(D–M)** Immunohistochemical localization of members of the TNF-system, IDO1, and AIF1 in the canine uterus at selected stages of early pregnancy. Signals of TNFα were present in macrophages during the pre-implantation **(D)** and implantation **(E)** periods. Similarly, both TNFR1 **(F)** and TNFR2 **(G,H)** were present in macrophages. In addition, weaker signals for both receptors were also observed in epithelial cells of uterine glands during pre-implantation **(F,G)** and in endothelial cells **(F,H)**. Positive signals of IDO1 were detected in macrophages between superficial glands during pre-implantation **(I)** and were also detected in endothelial cells at the time of implantation **(J)**. At post implantation, weaker signals were observed in macrophages localized in deep endometrium layers, as well as in endothelial cells **(K)**. AIF1 positive signals were identified in macrophages close to superficial uterine glands in pre-implantation **(L)** and implantation **(M)** stages, while at post-implantation they were localized in the deep layer of the endometrium **(N)**. Some weak signals were also observed in uterine glands at the pre-implantation period **(L)**, while apparently stronger signals were observed in the same glands at implantation **(M)** and post-implantation **(N)**. Weak positive signals were also detected in endothelial cells **(M**, **N)** (solid arrow = macrophages; closed arrowhead = uterine gland; open arrowhead = blood vessel). No staining is observed in the isotype controls [inset in **(D,F,G,I,L)**].

In addition to cytokines, the presence of other factors involved in the uterine response to early pregnancy was assessed (*TLR4, IDO1*, and *AIF1*, Figures 4A–C). Stage- or embryo-dependent effects were observed in all three factors: *P* < 0.01 for *TLR4*, and *P* < 0.05 for *IDO1* and *AIF1*. The mRNA availability of *TLR4* decreased dramatically and was the lowest post-implantation, when compared with all earlier stages (*P* < 0.05, Figure 4A). In contrast, *IDO1* and *AIF1* responded positively to the presence of free-floating embryos (*P* < 0.05, Figures 4B,C); their expression was, however, not further affected during the establishment of early pregnancy (*P* > 0.05).

The localization of different factors involved in immune regulation, i.e., members from the TNF-system, IDO1 and AIF1, was further evaluated (Figures 4D–N). TNFα-positive signals were observed in cells identified as macrophages in the superficial layer of the endometrium in pre-implantation (E+) and Imp samples (Figures 4D,E), with some rare cells being localized in the myometrium (not shown). As for its receptors, positive signals for TNFR1 could be identified in immune cells localized around superficial glands, with weaker signals also being visible in endothelial cells and glandular epithelial cells (Figure 4F). Regarding TNFR2, immune cells also presented positive signals in E+ and Imp samples (Figures 4G,H). However, only at the time of implantation could positive staining of the endothelium be observed (Figure 4H). Positive signals of IDO1 could be found during the pre-implantation phase in macrophages localized close to the luminal surface of the endometrium (Figure 4I). At the time of implantation, additional signals for IDO1 were present in some single cells localized within the myometrium (not shown) as well as, to a lesser extent, in endothelial cells (Figure 4J). Despite a similar localization pattern in Post-Imp samples, with IDO1 signals being present in macrophages and endothelial cells, positive signals appeared to be weaker at this stage and macrophages were mainly localized around deep uterine glands (Figure 4K) and within the connective tissue of the myometrium (not shown). As for AIF1, positive signals during pre-implantation were observed in macrophages localized close to superficial uterine glands and in the epithelial cells of these glands (Figure 4L). At the time of implantation, positive signals in glandular epithelium appeared stronger than in pre-implantation (Figure 4M). In addition, a low number of positively stained macrophages was still observed around the superficial glands and some weaker signals were detected in endothelial cells (Figure 4M). Finally, in Post-Imp samples, weak signals were observed in macrophages around deep glands (Figure 4N), with positive staining also detected in the epithelium of these deep glands and in endothelial cells (Figure 4N).

### IGFs and Markers of Tissue Remodeling

**Figure 5:**
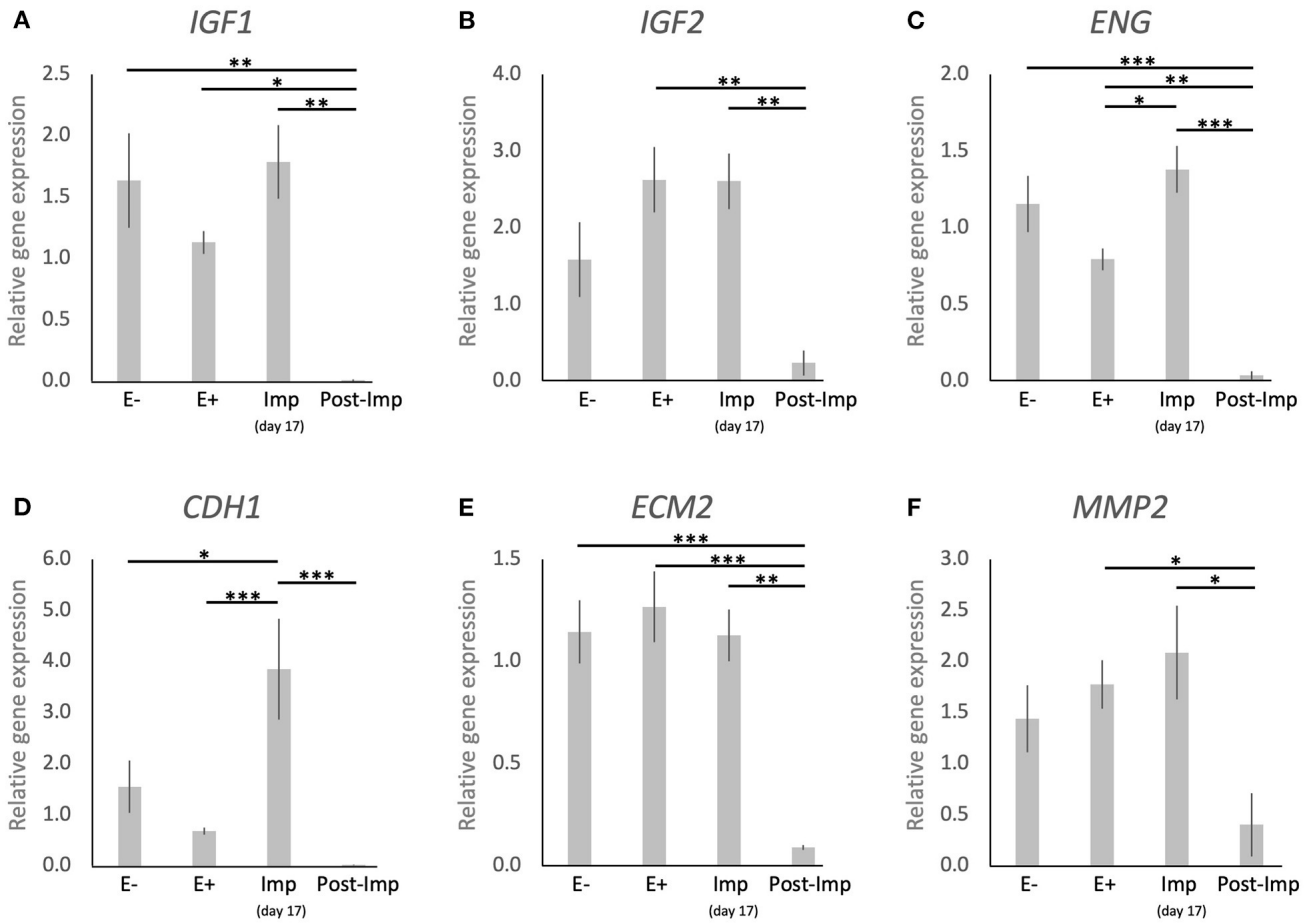
Relative gene expression of selected factors involved in tissue remodeling in the canine uterus. **(A–F)** Relative gene expression as determined by semi-quantitative real time (TaqMan) PCR (mean ± SEM). One-way ANOVA was applied, revealing: *P* = 0.0007 for *IGF1, P* = 0.0032 for *IGF2, P* < 0.0001 for *ENG, P* = 0.0002 for *CDH1, P* < 0.0001 for *ECM2, P* = 0.0174 for *MM2*. In the case of *P* < 0.05, this was followed by a Tukey-Kramer multiple comparisons post-test. Bars with asterisks differ at: *P < 0.05, **P < 0.01, ***P < 0.001.

The uterine expression of selected factors acting as growth factors or involved in tissue remodeling was evaluated (Figure 5). Time-dependent effects were observed in the expression of *IGF1* (*P* < 0.001), *IGF2* (*P* < 0.01), *ENG* (*P* < 0.0001), *CDH1* (*P* < 0.001), *ECM2* (*P* < 0.0001), and *MMP2* (*P* < 0.05). These were related predominantly to the stages of pregnancy as none of these factors were significantly modulated in response to the presence or absence of an embryo between days 8–12 (E– vs. E+). Interestingly, following the significant induction of *ENG* and *CDH1* in response to embryo attachment (E+ vs. Imp, *P* < 0.05 and *P* < 0.001, respectively; Figures 5C,D), the expression of all factors was significantly reduced after initiation of invasion and placentation (Post-Imp) (*P* < 0.05, Figures 5A–F).

## Discussion

Considering the importance of the immune system in conceptus-maternal communication during the establishment of pregnancy, highlighted in several species, the lack of knowledge regarding the dog is striking. This relates not only to the characterization of local immune signaling, but also to the absence of information regarding the immune cell population present in the uterus during pregnancy. Here, by investigating the expression and/or localization of selected markers in the dog uterus, we aimed to determine the presence of different subsets of macrophages and T lymphocytes known to play a role in the establishment of pregnancy in other species. The evaluation of the expression of different cytokines and growth factors was aimed at further characterization of the uterine immune milieu, addressing the functional dynamics between the implanting conceptus and maternal structures.

The differentiation between subsets of macrophages and lymphocytes presents several challenges, with different phenotypes expressing similar surface markers. Thus, the selection of a range of factors, as presented in Table 3, is required for such differentiation. Macrophages can polarize into M1 (classical activation, involved in the pro-inflammatory Th1 response) and M2 (alternative activation) phenotypes, with the latter presenting M2a (involved in the Th2 immune response), M2b (involved in pro-inflammatory responses and immune regulation), or M2c (tissue repair/remodeling) characteristics (6, 32–35). Both CD86 and MHCII are expressed in M1 and M2b macrophages, although the latter have a lower expression of MHCII (32, 33). Thus, while the evaluation of CD86 gene expression would not have been useful in validating the presence of each of these two different phenotypes, the immunohistochemical detection of MHCII allowed their differentiation. Furthermore, the expression of CD206 and CD163 was also investigated targeting the evaluation of macrophages with M2a and M2c functions (32). With regard to lymphocytes, the expression of CD8 by cytotoxic T cells is widely recognized, while NK cells can be identified by their production of NKp46 (encoded by NCR1) (36). Furthermore, both activated helper (Th) and regulatory (Treg) T cells phenotypes express CD4 and CD25 (37–40). Thus, the detection of FoxP3, a specific marker of Treg cells, was further required for differentiation among these cell populations (40). The evaluation of several immune factors that can be associated with different immune cell subsets (e.g., the expression of TLR4 and CCR7 by M1 macrophages) further substantiated the present analysis.

In agreement with previous findings (14, 17, 18), the presence of the embryo was associated with the modulation of the uterine immune milieu during the pre-implantation period (E+). Among the factors evaluated, the expression of *MHCII, CD4, CD25*, and *NCR1* was upregulated, while lower expression of *FoxP3* was observed. MHCII, which is essential for antigen recognition by T cells, is expressed in a wide variety of antigen presenting cells, including macrophages, monocytes, and dendritic cells. Its expression in other endometrial cells, like epithelial and stromal cells, has been reported in some species, e.g., in humans and rodents (41, 42), but in the present study positive signals were restricted to macrophages localized within the endometrial stroma. Interestingly, these MHCII-positive signals were colocalized with the cellular distribution of CD86-positive cells, suggesting that the increased expression of *MHCII* in the pre-implantation period, in contrast to E- and Post-Imp, appears to be associated with an increased infiltration of macrophages with M1 characteristics. As for lymphocytes, the increased expression of *NCR1*, a specific marker of NK cells, suggests an increased infiltration of these cells in response to the presence of the embryos. The potential importance of NK cells during early pregnancy will be addressed below.

Furthermore, the increased availability of *CD4* and *CD25*, accompanied by a decreased expression of *FoxP3*, a specific marker of Treg cells (40), appears to indicate an increased presence of Th cells in the uterus prior to implantation. This was further supported by the significantly higher expression of *CD4* than of the cytotoxic *CD8* (43). Even though the discrimination between Th subpopulations was not performed in the present work, the apparently concomitantly increased abundance of M1 and NK cell markers in the pre-implantation period suggests the presence of a dominant Th1 immunity in response to embryo presence. Pre-implantation was also marked by increased expression of the anti-inflammatory *IL10* and a decreased expression of the chemoattractant *CCL3*. Adding to our observation of a lower expression of *CD8* than *CD4*, this might be related to the presence of local immunomodulatory signals involved in the immunotolerance toward the embryo. Moreover, the canine embryo could be also involved in modulating the uterine immune response through the expression of factors like prostaglandin synthase 2 (PTGS2/COX2), PGE2 synthase (PTGES), and IGFs, as shown previously (15). In particular, PGE2 appears to be of importance for modulating the uterine immune milieu by being associated with suppression of cytotoxic activities of local immune cells [reviewed in (44)]. Furthermore, uterine-derived signals might also be involved in this immunomodulatory process. As also shown in our previous study, both *IDO1* and *AIF1* were upregulated in response to embryo presence (14). IDO1 plays a crucial role in prevention of immune-driven fetal rejection in the mouse (45). By controlling tryptophan degradation, this enzyme regulates leukocyte activation and is involved in a plethora of immunomodulatory mechanisms, i.e., decreasing NK cells cytotoxic activity, promoting Treg cell activation while inhibiting the functions of other T cell subsets and promoting the conversion of M1 macrophages to the M2 phenotype (46–49). In contrast, the role of AIF1 in the uterus is still poorly understood, but it has been associated not only with immunomodulatory processes, but also with proliferative and vascular mechanisms in several systems (50–53). Thus, IDO1 and AIF1 might be involved in the modulation of immune response in the pre-implantation period. In addition to both factors being localized in macrophages, IDO1 positive signals were also present in the endometrial endothelium during implantation and AIF1 expression was observed in epithelial glandular cells. Based on this, the modulation of the inflammatory signaling observed in the pre-implantation period appears to involve different uterine cell populations.

In contrast with the pre-implantation period, *MHCII* and *TLR4* were strongly downregulated during early placentation. Similarly, *CCR7* together with *CD4* were suppressed toward implantation and early placentation, cumulatively suggesting a decrease in M1 activity. This decrease in M1 activity following the establishment of pregnancy appears to reflect the situation described previously for other species, like the human and cow, where a shift between M1 and M2 activity is observed following placentation (6, 54). In humans, the decrease in M1 activity appears to be crucial for the maintenance of pregnancy, as the imbalance in the M1/M2 macrophage population is associated with an inadequate remodeling of uterine vascularization and spontaneous abortion [reviewed in (6, 38)]. Following this line, in the dog, implantation and, thus, early decidualization, was also marked by upregulated expression of *FoxP3*. This suggests an increased activity of the immunosuppressive Treg cells, even more strongly implying the functional transition from a proinflammatory to a modulatory immune reaction to embryo presence, attachment, and the ongoing morpho-functional remodeling of the uterus. While the full understanding of the role of Treg in the uterus is still missing, a decreased number of these cells is associated with recurrent abortions and preeclampsia in humans, and their depletion in mice leads to pregnancy loss (38, 39, 55). Thus, the presence of immunosuppressive Treg cells appears to be crucial in embryo-maternal contact and could also apply to canine reproduction. With regard to the regulation of this immune population, PGE2 increases the expression of FoxP3 in human peripheral CD4+CD25+ mononuclear cells (56). Thus, by expressing PTGES (15), the implanting canine embryo might be responsible for a local increase of PGE2 that could be involved in this increased presence of Treg cells. Furthermore, IL12a is described as being upregulated in actively suppressing Treg cells (57, 58), while IL6 can inhibit Treg cell activity and FoxP3 expression (59). Thus, considering that *IL12a* presented its highest expression during implantation, while *IL6* was downregulated, it appears plausible that these interleukins might be involved in the regulation of Treg cell presence in the uterus at the time of implantation. Furthermore, besides modulating Treg activity, IL12a is also involved in the activation of NK cells (60, 61). As mentioned elsewhere, these cells play a key role in the development of the decidua in humans and mice, mainly by modulating blood vessel development (7, 8). Following the increased expression of *NCR1* observed here in the pre-implantation and implantation periods, there appears to be an increased number of NK cells in the uterus during the establishment of canine pregnancy. Furthermore, the localization of NK cells close to uterine blood vessels in the superficial layer of the endometrium during pre-implantation and implantation might also suggest the involvement of this population in the modulation of uterine vascularization and decidualization as described in other species presenting decidua (7, 8). However, this hypothesis still needs to be verified for the dog.

Regarding the factors involved in uterine remodeling, *ENG* and *CDH1* became upregulated at the time of implantation compared with their expression in the pre-implantation uterus. ENG acts as a TGFβ receptor and, in the murine uterus, is associated with uterine receptivity for the implanting embryo (62). CDH1, that encodes for E-cadherin, is an important factor in cell adhesion and is involved in the functional modulation of endometrial morphology and implantation in several species [reviewed in (63)]. Thus, the increased expression of these factors implies their involvement in the canine implantation process.

Finally, the post-implantation period was marked by decreased availability of markers of M1 (*MHCII, TLR4*) and M2a (*CD206, IL10*) macrophages, T cells (*CD4*), NK cells (*NCR1*), and an apparent decrease of the marker of Treg cells *FoxP3*, in addition to other cytokines (*IL8, IL12a, TNFR1*). Although the quantification of immune cells in the uterus was not within the scope of the present work, these expression patterns suggest a decreased immune activity in the uterus during this period, despite the significantly increased expression of the pro-inflammatory *IL1*β. We found it interesting that the localization of CD206, CD163, and NKp46 positive cells was predominantly associated with the superficial uterine compartments during pre-implantation, contrasting with their increased presence in deeper endometrial layers following early placentation, i.e., during the post-implantation stage. It appears that not only the composition of the uterine immune population is affected with the progression of pregnancy, but also its localization in the uterus appears to change. These effects imply the presence of immunosuppressive signals possibly required to allow the invasion of the trophoblast during the placentation process. Furthermore, the expression of growth factors (*IGF1, IGF2*) and markers of tissue remodeling (*ENG, CDH1, ECM2*, and *MMP2)* was decreased during the post-implantation stage, at the time where the development of the placenta is accompanied by significant tissue remodeling. Nevertheless, considering that the samples analyzed in this study were derived from implantation sites, the decreased expression of factors involved in tissue growth and remodeling might actually reflect the proteolytic activity of the trophoblast over the endometrium during invasion.

### Conclusions

**Figure 6:**
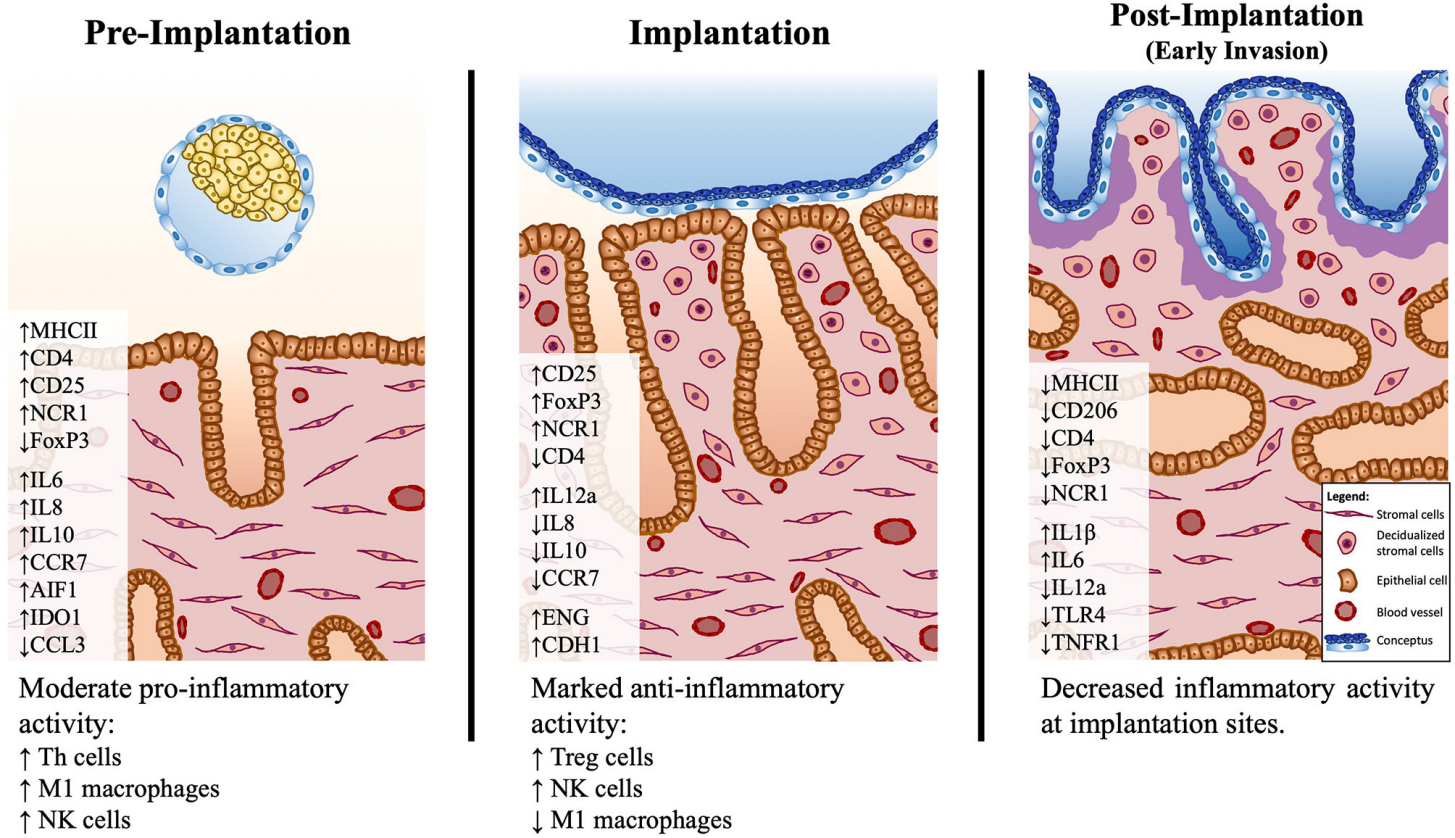
Schematic presentation of the proposed model of immune system–mediated events in the canine uterus during establishment of pregnancy. Arrows (↑ and ↓) indicate increased or decreased expression, respectively, of different selected factors during establishment of pregnancy: pre-implantation (days 10–12), implantation (day 17 of embryonal life), and post-implantation (day 25 of pregnancy). The presence of the free-floating embryo during pre-implantation is associated with increased inflammatory activity, marked by upregulated expression of markers of M1 macrophages (*MHCII*) and Th lymphocytes (*CD4, CD25*). This reaction appears to be moderated by the increased expression of immunomodulatory factors like *IDO1, AIF1*, and *IL10*. There is a shift in the immune milieu at the time of implantation, with decreased transcripts of *MHCII* and increased transcripts of Treg markers (*FoxP3*). Both pre-implantation and implantation stages show increased expression of the NK cell marker *NCR1*. During implantation, concomitantly, the first morphological signs of decidualization are observed in subepithelial endometrial layers. The contribution of the immune system-derived factors to the decidualization process needs further clarification. In the post-implantation period, the expression of several factors investigated is decreased at the implantation sites, indicating a decreased local immune activity in response to embryo invasion.

The evaluation of several immune cell-specific markers and other immune factors provided new insights into the uterine immunological status and possible functional dynamics during the early stages of canine gestation (summarized in Figure 6). The presence of the embryo clearly modulated the uterine milieu, inducing a controlled pro-inflammatory signaling in the pre-implantation period. This early stage appears to be under the influence of Th cells, that prevail over cytotoxic lymphocytes, accompanied by an increased presence of macrophages with proinflammatory M1 characteristics. Furthermore, the increased presence of NK cells during the pre-implantation and implantation periods suggests the involvement of this population in endometrial remodeling. Interestingly, Treg cells appear to have an important role at the time of implantation, probably being involved in the suppression of immune responses toward the invading embryo. To which extent the immune system-derived factors contribute to the concomitantly occurring canine-specific decidualization, remains to be investigated. Post-implantation proteolytic activity of the early invading trophoblast at implantation sites appears to be associated with locally decreased immune activity accompanied by lowered expression of IGFs and factors involved in tissue remodeling. In the modulation of immune responses, local factors like IDO1 and AIF1, derived from different uterine cellular components, and embryo-derived factors like PGE2 might be involved. Still to be considered is the species-specific uterine exposure to high circulating P4 levels. The immunosuppressive properties of P4 have been described in several mammals, including humans and rodents [reviewed in (64)]. Thus, the potential role of P4 in modulating local uterine immune responses in the dog appears plausible and should be taken into consideration in future research. Finally, the increased presence of NK and Treg cells in the pre-implantation and/or implantation stages implies similarities between the canine uterine immune milieu and the situation observed in humans and rodents [reviewed in (7, 8, 39)]. In fact, in our previous microarray paper, a higher correlation of embryo-induced effects in the uterus was observed between the dog and humans than with other domestic mammals (14). Such similarities appear to be further linked to the preparation of the uterus for the formation of the decidua and, possibly, also for the placentation. However, despite sharing the common reproductive goals of avoiding embryo rejection and successful implantation, and taking into account the restricted (shallow) invasion of the trophoblast during the formation of the canine endotheliochorial placenta, there may be species-specific regulatory features related to the local immune response in the dog.

## Data Availability Statement

The raw data supporting the conclusions of this article will be made available by the authors, without undue reservation.

## Ethics Statement

The animal study was reviewed and approved by Justus-Liebig University Giessen, Germany (permits no. II 25.3-19c20-15c GI 18/14 and VIG3-19c-20/15 GI 18,14); University of Ankara, Turkey (permits no. Ankara 2006/06 and 2008-25-124). Written informed consent was obtained from the owners for the participation of their animals in this study.

## Author Contributions

MTP and RN were involved in developing the concept of the present study, experimental design, generating data, analysis and interpretation of data, and drafting of the manuscript. RP-C, SM, SA, and DK were involved in the collection of tissue material, knowledge transfer, critical discussion and interpretation of data, and revision of the manuscript. MPK designed and supervised the project, and was involved in interpretation of the data, and drafting and revision of the manuscript. All authors read and approved the final manuscript.

## Conflict of Interest

The authors declare that the research was conducted in the absence of any commercial or financial relationships that could be construed as a potential conflict of interest.
